# From model failure to system harm: operationalizing a sociotechnical pathway for healthcare AI safety

**DOI:** 10.3389/frai.2026.1956533

**Published:** 2026-09-14

**Authors:** Burhan Sebin, Irem Karaman Sebin

**Affiliations:** 1 eMerge Americas, Miami, FL, United States; 2 Baptist Health South Florida, Miami, FL, United States; 3 Herbert Wertheim College of Medicine, Florida International University, Miami, FL, United States

**Keywords:** algorithmic bias, artificial intelligence, automation bias, clinical decision support, large language models, patient safety, sociotechnical systems

## Abstract

Healthcare artificial intelligence (AI) is commonly evaluated using measures of discrimination, calibration, sensitivity, specificity, and benchmark accuracy. Although these metrics are necessary, safety ultimately depends on what occurs after an AI output enters a clinical system, whether it is noticed, trusted, verified, acted upon, and propagated through existing workflows and organizational capacities. Evidence from postmarket reports, human-AI studies, drift analyses, and equity audits demonstrates that hazards can arise at multiple points along this pathway, while remaining insufficient to quantify the incidence, attributable severity, or long-term consequences of AI-related harm. We therefore propose a five-stage sociotechnical pathway that traces risk from upstream vulnerability through AI behavior, human-workflow mediation, decision or system effect, and downstream harm. Rather than adding another catalog of governance principles, the framework follows how a specific vulnerability propagates and treats each transition as an auditable control point linked to measurable indicators, accountable actors, escalation criteria, and response actions. Thresholds should be prespecified according to the intended use and local context.

## 1 Introduction

Artificial intelligence is increasingly embedded in diagnosis, screening, treatment planning, documentation, triage, risk stratification, resource allocation, and other aspects of healthcare operations. Yet, the language used to evaluate these systems remains largely model-centric. Measures of discrimination, calibration, sensitivity, specificity, and benchmark accuracy describe whether a model performs as intended under defined conditions; they do not determine what happens after its output enters clinical care. Reporting guidance has progressively expanded evaluation to include live clinical studies and trials (Cruz Rivera et al., 2020; Liu et al., 2020; Vasey et al., 2022), while governance and regulatory frameworks increasingly emphasize ethical deployment and lifecycle risk management (Lekadir et al., 2025; U.S. Food and Drug Administration, 2025; World Health Organization, 2021). These developments broaden the scope of evaluation, but they also highlight a central problem: technical performance is only one component of safety.

Once an AI output enters clinical care, it becomes one input in a sequence of human and organizational decisions. A false-positive result may be recognized and corrected without consequence, or it may trigger imaging, biopsy, referral, treatment, and anxiety, while consuming limited clinical capacity. Conversely, a technically correct recommendation may still cause harm if its threshold overwhelms referral capacity, its interface encourages inappropriate reliance, or its implementation shifts verification tasks to clinicians who lack sufficient time or authority. Thus, model errors do not necessarily result in patient harm, and technically correct model outputs do not necessarily ensure patient safety. The clinically relevant question is therefore not merely whether a prediction was correct, but how that prediction was interpreted, acted upon, and mediated by the surrounding clinical and organizational system.

This distinction is consistent with a longstanding body of healthcare informatics and human-factors research that conceptualizes digital technologies as components of sociotechnical systems rather than as isolated tools (Holden et al., 2013; Sittig and Singh, 2010). AI-specific frameworks have extended this perspective to contextual design and end-to-end implementation (Salwei and Carayon, 2022; van der Vegt et al., 2023). At the same time, the governance landscape has expanded rapidly: a 2026 scoping review identified 77 organizational frameworks (Wang A. et al., 2026), while recent approaches address real-world deployment (You et al., 2025), health-system implementation and review (Wells et al., 2025), implementation determinants (Powis et al., 2026), governance maturity (Hussein et al., 2026), meaningful human oversight (van de Sande et al., 2026), and the institutional operationalization of the European Union (EU) AI Act (Gehin et al., 2026). Together, this literature demonstrates that safe AI use depends not only on model performance but also on context, people, workflow, organizational conditions, and lifecycle oversight.

However, an important practical analytic question remains unresolved: when a vulnerability arises, how should it be traced through the clinical system to determine whether it is amplified, intercepted, or ultimately translated into an adverse consequence? This perspective addresses that question. We first examine evidence demonstrating that AI-related risks can emerge from device behavior, human–AI interaction, workflow and organizational conditions, model drift, inequity, and generative AI vulnerabilities. We then develop an operational sociotechnical pathway that brings these observations together within a common structure for tracing risk and identifying points at which mitigation can occur. The aim is not to replace existing reporting or governance frameworks, but to provide a focused approach for connecting model behavior with what ultimately occurs in clinical care (Gehin et al., 2026; Hussein et al., 2026; Lekadir et al., 2025; Powis et al., 2026; Vasey et al., 2022; Wells et al., 2025; You et al., 2025).

## 2 From model failure to system harm

To address this question, we define the relevant unit of analysis as the pathway through which AI output enters and influences clinical care. The pathway comprises five analytical stages, summarized together with their corresponding mitigation gates in Figure 1, although real-world events may branch, recur, bypass stages, or be interrupted. First, an upstream vulnerability may arise from target definition, data acquisition, representation, measurement, model design, software change, distribution shift, or compromised inputs. Second, that vulnerability may be expressed in AI behavior as misclassification, omission, false confidence, unstable output, or an unsafe recommendation; even technically correct output can become hazardous when thresholds or intended use are poorly aligned with the clinical setting. Third, human-workflow mediation determines whether the output is accepted, questioned, delayed, ignored, or verified. Fourth, the resulting response may alter testing, treatment, referral, documentation, discharge, or resource allocation. Fifth, these downstream changes may culminate in harm, although risk can also be intercepted before harm occurs.

**Figure 1:**
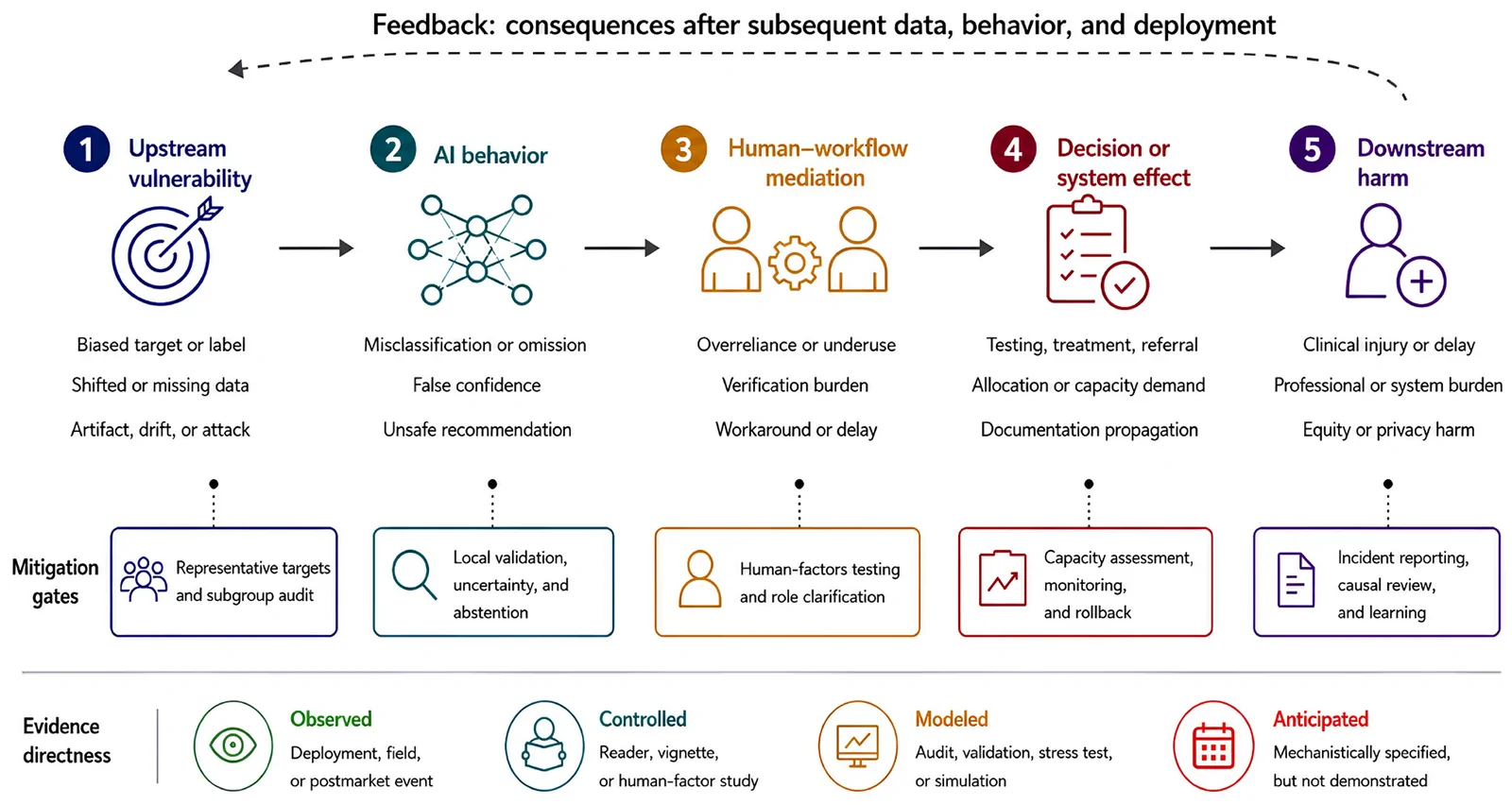
Pathway-based model of healthcare AI harm and corresponding mitigation gates.

Within this framework, downstream harm includes clinical injury or delay, inequitable access or treatment, privacy or security harm, and material professional or system-level burden when that burden degrades care. The pathway is therefore an analytical tracing device rather than a claim that every event follows a linear sequence or that AI output alone causes the final outcome. This approach is consistent with safety-science research that treats AI failures as sociotechnical events requiring analysis of how hazards interact with human and organizational controls and propagate through complex systems (Macrae, 2019, 2022). Although the framework is oriented toward harm analysis, the same transitions can also mediate benefits; monitoring should therefore capture both beneficial and adverse downstream effects.

The value of this distinction lies in its ability to avoid two opposite errors. The first is treating every technical failure as equivalent to patient injury: external validation, stress testing, and adversarial imaging studies can identify important hazards without establishing attributable harm in routine care (Schömig-Markiefka et al., 2021; Wong et al., 2021; Zhou et al., 2021). The second is assuming that technically correct output is necessarily safe: correct recommendations may still generate excessive referrals, verification burden, or inequitable allocation when they interact poorly with local thresholds and capacity. The purpose of the pathway is therefore to make these intermediate transitions visible.

## 3 Evidence across the pathway

If the pathway is clinically meaningful, evidence should be observable at multiple points along the trajectory from model behavior to patient outcome. The current literature does not provide a single denominator-based estimate of AI-related harm, but it does demonstrate many of these intermediate mechanisms through postmarket reports, controlled human studies, field implementations, longitudinal audits, equity analyses, and generative AI evaluations. We therefore organize the evidence according to where risk becomes visible and how far downstream each study follows the pathway.

### 3.1 Direct clinical and device-related consequences

Postmarket evidence shows that AI-enabled systems can be implicated in real-world adverse events but does not provide reliable estimates of their incidence. Among 266 reports involving machine-learning-enabled devices, 16% described actual harm, 9% described consequences for healthcare delivery, and 4% described near misses (Lyell et al., 2023); broader adverse-event and recall analyses likewise identified injuries, recalls, and device or software design problems (Chen et al., 2025; Lin et al., 2025). A 2026 lifecycle analysis of radiology AI devices approved by the U.S. Food and Drug Administration (FDA) found that 44.9% had undergone software-version updates and identified links between postmarket adverse events, recalls, and software defects (Li et al., 2026). Separately, 43 of 903 FDA-authorized AI-enabled devices (4.8%) were recalled in a 2026 cohort study (Ren et al., 2026). These studies provide evidence of occurrence and lifecycle relevance, but passive reporting lacks exposure denominators and rarely isolates the AI component from hardware, data transfer, workflow, and user-related factors.

### 3.2 Human–AI interaction

Controlled studies show that safe AI use depends on appropriately calibrated human reliance. In a randomized study of 457 clinicians, systematically biased AI reduced diagnostic accuracy by 11.3 percentage points, and image-based explanations did not significantly protect users from following incorrect advice (Jabbour et al., 2023). Reader and decision studies likewise demonstrate susceptibility to erroneous recommendations (Dratsch et al., 2023; Gaube et al., 2021; Kim et al., 2025; Kücking et al., 2024). A suppression strategy reduces model automation bias by selectively withholding some AI recommendations (Wang et al., 2023). A 2026 meta-analysis found task-dependent potential benefits from human-large language model (LLM) collaboration but substantial uncertainty across outcomes (Wang G. et al., 2026). Recent work on meaningful oversight further argues that clinician presence is insufficient without epistemic capacity, cognitive space, decisional authority, and effective opportunities for intervention (van de Sande et al., 2026). The safety target is therefore calibrated around reliance under realistic workload and error conditions, rather than reliance or non-reliance alone.

### 3.3 Workflow, organizational burdens, and drift

Workflow and lifecycle conditions can amplify moderate technical limitations. Field evaluations have documented delays, workarounds, redistributed authority, and uncertain benefit during AI implementation (Beede et al., 2020; Johnson et al., 2024). Alert acceptance declines with repeated notifications; triage can reduce physician alerts while transferring work elsewhere, and repeated computer-aided colonoscopy prompts have shown declining responsiveness across procedural sequences (Ancker et al., 2017; Dahmke et al., 2023; Huang et al., 2026). In a nationally deployed Veterans Health Administration model, positive predictive values declined, and false positives increased over time (Kolla et al., 2025); a longitudinal acute kidney injury prediction study found heterogeneous drift and showed that model updating could improve performance (Xu et al., 2025). Software version updates and postmarket events are also incompletely linked throughout the device lifecycle (Li et al., 2026). Monitoring that is limited to discrimination metrics may, therefore, miss changes in workload, delays, subgroup effects, and version-associated risks.

### 3.4 Equity and population-level harm

Equity risks may arise from biased targets, differential errors, or measurement pathways. A population-health algorithm using expenditure as a proxy for need systematically underestimated illness burden among Black patients (Obermeyer et al., 2019). Chest-radiograph classifiers and readmission models demonstrated subgroup disparities and context-dependent calibration (Seyyed-Kalantari et al., 2021; Wang et al., 2024), while fairness audits may themselves be limited by poor demographic data (Lu et al., 2022). Related measurement studies show the same propagation mechanism: race-based spirometry equations altered treatment recommendations (Visness et al., 2025), and pulse oximetry more frequently missed hypoxemia among Black patients (Sjoding et al., 2020). These latter examples are not AI systems; they illustrate how biased inputs, proxies, or correction rules can alter eligibility, urgency, and treatment when incorporated into algorithmic pathways.

### 3.5 Generative AI output hazards

Generative AI safety evidence is expanding but remains dominated by non-prospective designs. A 2026 review identified 4,609 peer-reviewed clinical LLM studies; only 1,048 used real-world patient data, and only 19 of those were prospective randomized trials (Chen et al., 2026). A separate evidence map of 55 intervention studies found heterogeneous clinical effects, inconsistent efficiency outcomes, and suboptimal reporting quality (He et al., 2026). In empirical evaluations, LLMs showed limitations in realistic diagnostic and management tasks (Hager et al., 2024), produced potentially harmful incorrect answers to oncology questions (Longwell et al., 2024), and demonstrated variable antibiotic-prescribing performance; the latter study also documented repetition of personal information supplied in prompts (Ngoc Nguyen et al., 2025). These findings establish credible output-level hazards but do not establish the frequency of patient injury during supervised deployment.

### 3.6 Deployment evidence

Deployment evidence illustrates why output hazards, clinician uptake, and patient outcome must remain distinct. In 16 Kenyan primary-care clinics, reviewers identified actively harmful LLM recommendations in 115 of 1,469 encounters (7.8%); 67 of these recommendations appeared in final documentation, while preexisting risk was fully mitigated in 118 encounters (8.0%) (Agweyu et al., 2026b). A subsequent pragmatic cluster-randomized trial of 9,691 patients found no significant difference in 14-day treatment failure and reported no serious adverse events deemed related to the intervention (Agweyu et al., 2026a). Together, these findings show why pathway monitoring should capture both beneficial and harmful effects and distinguish among model outputs, their uptake or correction, and downstream patient outcomes.

### 3.7 Security, privacy, and robustness vulnerabilities

Adversarial and privacy studies identify additional vulnerabilities that may emerge before or during deployment. Prompt injection and fabricated clinical facts can substantially alter model outputs (Lee et al., 2025; Omar et al., 2025). Partially synthetic data can remain vulnerable to membership inference, whereas fully synthetic data may be only marginally susceptible (Zhang et al., 2022). Curated retrieval can reduce hallucinations but introduces trade-offs in response characteristics (Nishisako et al., 2025). These findings reinforce the need to evaluate security, privacy, and robustness as pathway-level hazards, rather than inferring patient injury solely from output behavior.

## 4 What evidence can and cannot establish

Before considering how this evidence supports clinical risk, Table 1 summarizes where each evidence domain becomes observable along the pathway, how consequences were measured directly, and which assurance mechanisms it suggests.

**Table 1:** Representative evidence supports a pathway-based account of healthcare AI harm.

Harm domain	Representative evidence	Directness	Interpretation	Assurance mechanism	Responsible actor	Monitoring indicator
Clinical/device	Postmarket/recall/lifecycle evidence (Chen et al., 2025; Li et al., 2026; Lin et al., 2025; Lyell et al., 2023; Ren et al., 2026)	Observed	AI-enabled systems can be involved in injuries, delivery disruptions, recalls, and software-related lifecycle events; the incidence remains unknown.	Local validation; version traceability; incident response	Developer; organization; regulator	Harm/near miss; recall/version signals
Human–AI interaction	Human–AI experiments, synthesis, and oversight (Dratsch et al., 2023; Gaube et al., 2021; Jabbour et al., 2023; Kim et al., 2025; Kücking et al., 2024; van de Sande et al., 2026; Wang et al., 2023; Wang G. et al., 2026)	Controlled/anticipated	Incorrect advice can worsen decisions; clinician presence or explanations alone do not ensure calibrated reliance.	Incorrect advice/reliance testing; escalation	Developer; human factors; clinical leadership	Erroneous advice concordance; override; verification time
Workflow/lifecycle	Implementation, alert, drift, and lifecycle studies (Ancker et al., 2017; Beede et al., 2020; Dahmke et al., 2023; Huang et al., 2026; Johnson et al., 2024; Kolla et al., 2025; Li et al., 2026; Xu et al., 2025)	Observed/controlled	Implementation changes work and authority; alert burden, model drift, and software-version changes can alter downstream effects.	Workflow/capacity assessment; longitudinal monitoring	Organization; developer	Workload; delay; unresolved output; drift/version change
Equity/population	Proxy/fairness/measurement studies (Lu et al., 2022; Obermeyer et al., 2019; Seyyed-Kalantari et al., 2021; Sjoding et al., 2020; Visness et al., 2025; Wang et al., 2024)	Observed/modeled	Biased targets, measurements, or errors can alter classification, allocation, access, and treatment.	Target/label review; subgroup/access audit	Developer; organization; equity oversight	Subgroup errors; allocation; access; delay
Generative AI/security	Synthesis, clinical, deployment, and adversarial studies (Agweyu et al., 2026a,b; Chen et al., 2026; Hager et al., 2024; He et al., 2026; Lee et al., 2025; Longwell et al., 2024; Ngoc Nguyen et al., 2025; Nishisako et al., 2025; Omar et al., 2025; Zhang et al., 2022)	Observed/controlled/ modeled	Unsafe or attack-sensitive outputs are documented; patient-level incidence and severity remain context-specific.	Adversarial testing; grounding; abstention; verification	Developer; organization; clinical user	Harmful/unverified output; attack success; abstention

Taken together, these studies support a consistent but limited conclusion. Device malfunction, biased proxies, differential errors, automation bias, workflow burden, drift, and generative AI vulnerabilities have been demonstrated in observational, controlled, or modeled settings. What remains much less certain is how often these hazards result in attributable patient injury, because postmarket reports lack denominators, controlled studies often use vignettes or reader tasks, and retrospective audits commonly end with model or decision outcomes rather than downstream patient outcomes. The Kenyan deployment studies illustrate this distinction: harmful recommendations and their propagation into final documentation were observed retrospectively (Agweyu et al., 2026b), whereas a pragmatic trial found no significant difference in treatment failure and no intervention-related serious adverse events (Agweyu et al., 2026a). The evidence therefore supports recognizable, context-dependent pathways through which harm may occur, but not a universal estimate of AI-related clinical risk.

This uncertainty does not imply that safeguards should be deferred until incidence is known. Rather, it limits the strength of claims that can be made about their effectiveness. Long-term professional and system effects, including deskilling, cumulative algorithmic burden, opportunity cost, vendor dependence, and decommissioning, remain poorly quantified. Current consensus, regulatory, implementation, and institutional governance frameworks emphasize evaluation and monitoring throughout deployment (Gehin et al., 2026; Lekadir et al., 2025; Li et al., 2026; U.S. Food and Drug Administration, 2025; Wells et al., 2025; You et al., 2025), while the 2026 governance scoping review found limited real-world applicability for many frameworks and called for the evaluation of governance impact (Wang A. et al., 2026). Evidence that governance processes themselves reduce patient harm therefore remains limited. The controls proposed below should consequently be treated as testable safeguards grounded in demonstrated mechanisms, rather than as a validated safety standard.

## 5 A pathway-based safety architecture

If risk can be amplified or interrupted at multiple transitions, the safety architecture should place controls at those transition points rather than relying on a single predeployment performance threshold. We therefore translate the five-stage pathway into five corresponding gates. Each gate addresses a different operational question: whether the targets and data are valid; whether the model is locally reliable; whether users can exercise meaningful oversight; whether downstream capacity can absorb the model's effects; and whether incidents are reported in a form that supports learning. Example monitoring indicators, accountable actors, review cadences, escalation criteria, and response actions for these five stages are summarized in Table 2.

**Table 2:** Example operational controls for implementing the pathway-based safety architecture.

Stage/control point	Example monitoring indicators	Accountable actor(s)	Review cadence	Escalation criterion	Example response
1. Upstream vulnerability	Missingness; label disagreement; subgroup representation; measurement/provenance shift	Developer/data steward; clinical owner	Predeployment; after material change; scheduled audit	Validity-boundary breach; unexplained shift; material underrepresentation	Hold deployment; investigate; revise target/data/measurement; revalidate
2. AI behavior	Local performance; abstention/out-of-distribution (OOD) rate; subgroup errors	Model owner/developer; deploying organization	Silent test; scheduled monitoring; after model/data/version change	Local/subgroup boundary breach; unexpected out-of-distribution (OOD) or version deterioration	Restrict use; investigate; revalidate; rollback/suspend
3. Human-workflow mediation	Erroneous advice concordance; override; unresolved outputs; verification time; alert burden	Workflow owner; human factors/clinical leadership	Predeployment; early postdeployment; after workflow change	Unsafe reliance; unresolved outputs; burden beyond local tolerance	Redesign; clarify roles; adjust alerts; restrict and retest
4. Decision/system effect	Testing/referral/treatment; waiting time; utilization/workload; subgroup access/delay	Clinical service; operations; quality/safety	Baseline; early and scheduled surveillance; after threshold/volume change	Capacity breach; meaningful delay/utilization increases; subgroup disparity	Adjust threshold/workflow; add capacity; narrow indication; rollback
5. Downstream harm	Harm/near miss per exposure; severity/recurrence; downtime; privacy/security events	Safety/AI governance; system owner; developer	Real-time for serious events; periodic aggregate review	Sentinel/recurrent event; rate above tolerance; unresolved safety signal	Contain; adjudicate/report; correct; revalidate; rollback/withdraw

These examples are an operational template rather than a validated or universal safety standard. Specific metrics, review intervals, and escalation thresholds should be defined prospectively for intended clinical use and revised when the model, workflow, population, or deployment context changes.

### 5.1 Gate 1: validating the target, data, and measurement process

Safety begins before model training. Targets and proxies should be clinically justified, while data provenance, measurement validity, missingness, and subgroup representation should be evaluated before deployment and following any material change. Biased proxies can encode structural inequity even when a model performs well on conventional metrics (Obermeyer et al., 2019), and subgroup audits can reveal performance or calibration disparities that average metrics obscure (Lu et al., 2022; Seyyed-Kalantari et al., 2021; Wang et al., 2024). Related measurement studies further show how biased measurement rules can alter clinical action (Sjoding et al., 2020; Visness et al., 2025).

### 5.2 Gate 2: establish local validity and conditions for abstention

External validation should be complemented by local evaluation under the intended data, workflow, staffing, prevalence, and capacity conditions, with ongoing monitoring after deployment (Lekadir et al., 2025; Wells et al., 2025; You et al., 2025). Systems should define how uncertainty and inputs outside validated conditions are handled, including escalation or abstention when reliable inference is not possible. Material changes to the model, data, or software version should trigger proportionate revalidation (Li et al., 2026; U.S. Food and Drug Administration, 2025).

### 5.3 Gate 3: test human reliance and workflow ownership

Human factors evaluation should test responses to incorrect AI advice (Dratsch et al., 2023; Gaube et al., 2021; Jabbour et al., 2023; Kim et al., 2025; Kücking et al., 2024) and to selectively withheld recommendations (Wang et al., 2023). Outcomes of task-dependent human-LLM collaboration also vary across studies (Wang G. et al., 2026). Verification, override, escalation, and documentation responsibilities must be explicit because implementation and alert studies show that interface burden, repeated notifications, and redistribution of verification work alter user responses (Ancker et al., 2017; Beede et al., 2020; Dahmke et al., 2023; Huang et al., 2026; Johnson et al., 2024). Meaningful oversight additionally requires epistemic capacity, cognitive space, decisional authority, and intervention effectiveness (van de Sande et al., 2026).

### 5.4 Gate 4: evaluate downstream capacity and monitor consequences

Organizations should model how decision thresholds may change testing, referral, treatment, documentation, workload, and shared capacity before deployment, and then monitor those effects after launch. For example, a threshold that improves case detection may still degrade care if the resulting referral volume exceeds specialist or diagnostic capacity, thereby lengthening waiting times. Monitoring should therefore link outputs to actions, waiting times, utilization, subgroup access, and patient outcomes. Capacity breaches or meaningful delays should trigger prespecified responses, such as threshold adjustment, workflow redesign, added capacity, narrowing the indication, or rollback, with downtime procedures defined in advance.

### 5.5 Gate 5: report incidents in a form that permits learning

AI-related incident reports should, under our proposed schema, identify the product and version, intended use, clinical setting, input state, output and uncertainty, user response, downstream action, event severity, affected subgroup, corrective action, recurrence, and, where definable, an exposure denominator. Postmarket studies show that event counts lack reliable exposure denominators and often cannot isolate the AI component's contribution (Lin et al., 2025; Lyell et al., 2023), while recall and lifecycle analyses illustrate the importance of linking product and software-version histories to safety events (Chen et al., 2025; Li et al., 2026; Ren et al., 2026). These elements are intended to support incidence estimation, cross-system comparison, and causal learning, rather than to imply that every event is attributable to AI.

## 6 Research and governance priorities

The major evidence gap identified above is not the absence of documented hazards, but the lack of studies that trace those hazards across the complete pathway to patient and system-level outcomes. Prospective evaluation should therefore connect AI model outputs with clinician responses, downstream actions, resource use, subgroup effects, and patient outcomes. This gap is particularly visible in LLM research, where evidence remains dominated by simulated or non-prospective evaluations (Chen et al., 2026) and intervention studies are heterogeneous and incompletely reported (He et al., 2026); the Kenyan cluster-randomized trial demonstrates that pragmatic, prospective evaluation at scale is feasible (Agweyu et al., 2026a). Priority designs include prospective registries of AI-associated safety events; interrupted time-series studies of downstream demand; longitudinal studies spanning model versions; repeated-measures studies of clinician performance; subgroup analyses of access, action, delay, and outcomes; electronic health record surveillance of propagated documentation errors; and planned downtime or decommissioning studies. Null and beneficial outcomes should be reported alongside adverse outcomes to ensure that safety is interpreted within an explicit benefit-risk framework.

The same logic applies to governance. Developers retain responsibility for target validity, data provenance, version control, and characterized limitations; healthcare organizations are responsible for local validation, workflow design, capacity assessment, monitoring, and incident response; and clinicians require usable uncertainty information, authority to override, and adequate time for verification. Recent frameworks provide practical structures for real-world deployment and review (Wells et al., 2025; You et al., 2025), implementation evaluation (Powis et al., 2026), governance maturity (Hussein et al., 2026), meaningful human oversight (van de Sande et al., 2026), and institutional operationalization of the EU AI Act (Gehin et al., 2026). The 2026 scoping review nonetheless found that many governance frameworks lack real-world applicability or comprehensive oversight mechanisms (Wang A. et al., 2026). The next empirical question is therefore not whether governance structures can be specified, but whether their implementation reduces routine safety events and patient harm.

## 7 Scope and limitations

This perspective is a thesis-driven synthesis rather than a systematic or scoping review. Literature was purposively selected to represent complementary evidence across postmarket safety, deployed systems, human–AI interaction, workflow, drift, equity, generative AI safety, prospective deployment, and governance. Priority was given to studies that demonstrated a component of the pathway, linked model behavior to human or system consequences, or informed an assurance mechanism. No formal search, screening, risk-of-bias assessment, or evidence-grading protocol was applied; claims about evidence strength therefore refer to the relative directness of the evidence rather than formal certainty. Some measurement studies are included only to illustrate propagation mechanisms, and several cited studies establish a hazard or mechanism rather than an attributable injury.

The operational template has not been prospectively validated and does not provide universal numerical safety thresholds. Acceptable error, alert, abstention, delay, or incident rates depend on intended use, baseline risk, alternatives, staffing, and downstream capacity. Local thresholds must therefore be prespecified and prospectively tested. Generative AI findings may evolve, but the pathway is intended to remain technology agnostic.

## 8 Conclusion

Healthcare AI safety cannot be inferred from model accuracy alone because harm is produced or prevented as AI outputs move through a sociotechnical system. The literature reviewed here identifies hazards at multiple points in that process, including postmarket failures, biased allocation and classification, automation bias, workflow burden, drift, and generative AI vulnerabilities. The evidence is substantially less complete regarding attributable patient outcomes, incidence, long-term professional effects, and the effectiveness of safeguards. The practical implication is a shift from model performance to pathway accountability: evaluation should follow how an output enters a specific clinical system, where risk is amplified or intercepted, who owns each transition, what is monitored, and what triggers escalation, restriction, rollback, or withdrawal.

Our proposed pathway makes this chain explicit and converts its transitions into points that can be evaluated, monitored, and acted upon. Healthcare AI safety should therefore be treated as a lifecycle property of the sociotechnical system rather than a static attribute of the model. Prospective deployments that measure the chain from AI exposure to human response, downstream action, resource effect, and patient outcome are needed to quantify risk and determine which safeguards effectively prevent harm.

## Data Availability

No original datasets were generated or analyzed for this article. All information supporting this perspective is contained within the article and the cited literature.
